# A Flexible Multi-Channel Deep Network Leveraging Texture and Spatial Features for Diagnosing New COVID-19 Variants in Lung CT Scans

**DOI:** 10.3390/tomography11090099

**Published:** 2025-08-27

**Authors:** Shervan Fekri-Ershad, Khalegh Behrouz Dehkordi

**Affiliations:** 1 Department of Computer Engineering, Na.C., Islamic Azad University, Najafabad 8514143131, Iran; khalegh.behrouz@iau.ir; 2 Big Data Research Center, Na.C., Islamic Azad University, Najafabad 8514143131, Iran

**Keywords:** convolution neural network, texture image analysis, deep network, feature extraction, computer tomography scan

## Abstract

Background: The COVID-19 pandemic has claimed thousands of lives worldwide. While infection rates have declined in recent years, emerging variants remain a deadly threat. Accurate diagnosis is critical to curbing transmission and improving treatment outcomes. However, the similarity of COVID-19 symptoms to those of the common cold and flu has spurred the development of automated diagnostic methods, particularly through lung computed-tomography (CT) scan analysis. Methodology: This paper proposes a novel deep learning-based approach for detecting diverse COVID-19 variants using advanced textural feature extraction. The framework employs a dual-channel convolutional neural network (CNN), where one channel processes texture-based features and the other analyzes spatial information. Unlike existing methods, our model dynamically learns textural patterns during training, eliminating reliance on predefined features. A modified local binary pattern (LBP) technique extracts texture data in matrix form, while the CNN’s adaptable internal architecture optimizes the balance between accuracy and computational efficiency. To enhance performance, hyperparameters are fine-tuned using the Adam optimizer and focal loss function. Results: The proposed method is evaluated on two benchmark datasets, COVID-349 and Italian COVID-Set, which include diverse COVID-19 variants. Conclusions: The results demonstrate its superior accuracy (94.63% and 95.47%, respectively), outperforming competing approaches in precision, recall, and overall diagnostic reliability.

## 1. Introduction

Since the onset of the global COVID-19 pandemic over 40 months ago, the virus has had a devastating impact worldwide. Official World Health Organization (WHO) reports indicate that more than 800 million confirmed cases have been recorded, with approximately 8 million deaths attributed directly to the virus as of December 2022. Additionally, nearly 40% of survivors have experienced long-term physical and respiratory complications. COVID-19 remains clinically unpredictable, posing a persistent threat to healthcare systems by straining resources and reducing efficiency. A critical challenge in managing this pandemic and future infectious disease outbreaks is the lack of rapid and accurate diagnostic tools [1].

**Figure 1:**
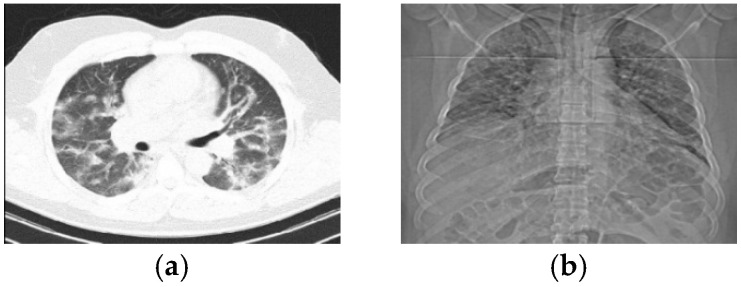
A lung image of a male person in two imaging types. (**a**) Lung CT-scan. (**b**) Chest radiography.

Rapid detection of positive cases and their quarantine are the most effective ways of controlling this epidemic. Thus far, various methods have been proposed to identify positive cases of the COVID-19 virus, some of which have been approved by the World Health Organization [2]. Reverse-transcription polymerase chain reaction (RT-PCR) is one of the most efficient diagnostic methods [3]. But, the false-negative rate of RT-PCR tests is relatively high, making it insufficiently efficient in communities with high transmission rates [4,5,6]. Also, the sensitivity of this test is low for detection during early stages when the level of infection is low [7]. There is also a contradiction between the lack of laboratory equipment and the rapid and accurate screening of suspected persons. These restrictions cause the loss of the critical time window for diagnosing patients, making it ineffective for preventing the widespread transmission of the virus and reducing the death rate. Researchers have used the combination of machine-learning and computer-vision techniques to analyze medical images, such as lung CT scans, which has provided acceptable results for the early diagnosis of patients with COVID-19. Computed tomography (CT) scans, like X-ray imaging, use photons to create images of internal tissues. Photons belong to the electromagnetic spectrum, along with visible light, radio waves, and microwaves, but X-ray photons have a higher energy value. CT images are defined as two-dimensional images that represent three-dimensional physical objects. These images are made by converting electrical energy (known as the movement of the electrons) into X-ray photons. As the photons pass through the object, the produced photons are converted back into electrons. The speed at which COVID-19 disease is diagnosed via an analysis of radiographic images is significantly higher compared to an RT-PCR test, but the examination of radiological images by specialists increases the workload of doctors during epidemics and is expensive. The identification of patients with COVID-19 depends on the experience of the radiologist and specialist doctor. Therefore, in recent years, to reduce the impact of individual biases, the use of automatic and intelligent methods has attracted the attention of researchers and medical equipment manufacturers. The diagnosis process in most intelligent medical diagnosis systems is fixed. With most tools, images in formats such as a PET or CT scan are given as input to the software, and then after the learning and decision-making process, the generated output is provided to the specialist doctor for the final decision. With diseases such as COVID-19, due to the high transmission rate, the importance of intelligent automatic diagnostic systems is much greater, because they can prevent direct contact between infected individuals and the radiologist, specialist, and other patients. The main goal of most related research is to increase the classification accuracy [8,9,10,11,12,13,14,15,16,17,18]. A large number of studies have proposed methods based on deep learning [11,12,13,14,15]. However, methods based on handcrafted features and machine-learning techniques have also exhibited acceptable results in some studies [16,17,18]. Studies show that the structure of the lungs in people infected with COVID-19, compared to healthy individuals, undergoes visible changes in texture and distribution of intensity. As an example, lung images of a 51-year-old male patient are shown in Figure 1, provided in two formats (CT scan and X-ray). The first research gap related to the problems associated with diagnosing patients with COVID-19 is the inability of machine-learning-based methods to detect new variants of this virus. In methods based on deep learning, due to network training, the above research gap is less pronounced. However, due to the focus on the spatial domain of the input image, these methods do not analyze lung texture and are inefficient for the detection of some types of COVID-19 that completely change the visual texture of the lungs, which is the second research topic within this scope.

Chest X-rays and lung CT scans are both imaging techniques, but they differ significantly in their detail. Chest X-rays provide a basic, two-dimensional (2D) image of the chest, while CT scans offer detailed, three-dimensional (3D) images of the lungs and surrounding structures. Chest X-rays (radiography) produce a 2D image by passing X-rays through the chest. X-rays provide less detail than CT scans and primarily useful for visualizing bones, heart, and large lung structures. Chest X-rays involve lower radiation exposure than CT scans. CT scan images create detailed 3D images of the chest by taking multiple X-ray images from different angles and using a computer to reconstruct them. The CT scan’s main advantages over a chest X-ray are its high detail, allowing visualization of small nodules or other abnormalities in the lungs and surrounding tissues or blood vessels. 

In this article, a machine-learning-based method for detecting COVID-19 is presented, which, unlike common methods in this family, does not have a feature engineering stage and uses deep features for classification. The method presented in this article includes three main stages: preprocessing, feature extraction, and classification. In the feature extraction stage, a two-channel deep convolutional neural network (DCNN) is used. Unlike common deep networks, the presented DCNN is fed in one channel with image texture features and in the other channel with visual information in the spatial domain. Layers related to classification, such as fully connected and softmax, have been removed from the internal structure and replaced with a flatten layer. Finally, a fine-tuned random forest classifier has been used. In order to increase the efficiency, both channels have been trained separately using relevant CT scan images, and their hyperparameters have been optimized using an Adam optimizer algorithm and a focal loss function.

The main goal of this article is to diagnose COVID-19 based on the analysis of lung CT scan images. The following two main contributions are presented in this paper:

- Using a two-channel convolutional neural network with the same structure in channels and two different power sources can increase the accuracy of COVID-19 detection compared to existing methods.
- When using a consistent and tuned classifier, the use of deep features in a classical machine-learning pipeline in which feature extraction and classification are performed separately can achieve higher accuracy than many popular deep neural networks for COVID-19 diagnosis.

The main novelty of this paper is the design of a two-channel deep convolutional neural network with two different input sources in such a way that one source carries the image information in the spatial domain, while the second source feeds the DCNN with image texture features extracted using a modified local binary patterns operator. Also, the proposed two-channel DCNN is only used for feature extraction and is applied for diagnosis of COVID-19 in a general machine-learning format.

In other words, the design of a two-channel deep neural network with two separate input sources, which enables all features to originate from the training process, unlike concatenation techniques, and the use of deep features of such a network in a larger structure based on the random forest classifier is the innovation of this paper, which has not been considered in previous research.

The rest of the paper is organized as follows: In Section 2, some related studies are discussed which used machine-learning or deep learning techniques for COVID-19 diagnosis. In Section 3, our proposed two-channel deep neural network is described with technical details. Section 4 presents the experimental results. Finally, the discussion and conclusion are included.

## 2. Related Works

As mentioned in the Introduction Section, many different methods have been presented to identify COVID-19 patients based on the analysis of lung CT scan images [19]. Most of the methods presented in this field are either based on classical machine-learning techniques or use deep learning techniques [20]. Most machine-learning-based methods include two stages of feature extraction and classification, which are implemented separately [21]. In the feature extraction stage, visual parameters of the image such as color, texture, and shape are usually analyzed. In some studies, the image is first transferred to another domain such as frequency domain or wavelet, and then handcrafted features or visual features are extracted in that domain. Less innovation occurs in the classification stage, and mostly supervised or unsupervised classifiers are usually used. Most recent studies have used deep learning-based techniques to diagnose patients with COVID-19. In deep neural networks, the process of feature extraction and classification is performed simultaneously and the two phases are not considered separately.

As mentioned in the Introduction, the method presented in this article is based on the structure of machine learning. Therefore, feature extraction and classification were performed separately. However, to increase the efficiency of the proposed method, a two-channel deep convolutional neural network is used in the feature extraction stage. In the rest of this section, some of the most relevant and efficient methods from these two groups are reviewed.

Ravi et al. [22] present an approach for COVID-19 detection in CT scan images based on a combination of meta-classifiers and deep neural networks. In order to extract deep features, the output of the last global average pooling layer in EfficientNet is used. Also, kernel principal component analysis (KPCA) is performed to reduce dimensions. Finally, a stacked ensemble meta-classifier-based approach was used for classification. A combination of random forest, support vector machine, and logistic regression is used in the two-step classification process [22].

Soni et al. [23] analyzed the performance of deep networks for COVID-19 detection in CT scan images. In [23], deep features were extracted from the final layers of different popular deep neural networks, which are fed into machine-learning-based classifiers. In their study, features were obtained using ResNet50, Inception V3, and EfficientNetB7. The results reported in [23] show that the combination of InceptionV3 and SVM achieved the highest classification accuracy.

Perumal et al. [24] proposed a method for COVID-19 detection using both lung X-ray and CT scan images. Their approach first extracts Haralick features from CXR and CT images firstly. Next, VGG16, ResNet50, and InceptionV3 were employed as backbones to extract deep features. Finally, transfer learning techniques were applied to improve classification models. Finally, numerical threshold values were proposed for certain features to classify diseases [24].

Santos and Melin [25] presented a new approach for COVID-19 recognition based on its manifestation on lung X-rays. In this regard, three different experiments were performed to classify lung X-ray images. In some experiments, handcrafted texture features were extracted using GLCM and local binary patterns (LBPs), combined with a feedforward multi-layer perceptron neural network. In another experiment, a deep convolutional neural network (DCNN) is used for the classification phase [25].

As previously mentioned, several researchers presented COVID-19 diagnosis methods based on classical machine-learning techniques.

Ozturk et al. [26] proposed a multi-stage method for COVID-19 detection from X-ray and CT scan images. In the first stage, shallow image augmentation was performed to balance the dataset and ensure sufficient data samples. Next, multiple sets of handcrafted features were extracted using gray-level co-occurrence matrix (GLCM), local binary patterns (LBPs), and segmented fractal texture analysis (SFTA). Subsequently, feature merging and data over sampling process were performed. In the pre-classification stage, feature reduction was performed using principal component analysis (PCA). Finally, an SVM was employed as a binary machine-learning classifier for COVID-19 diagnosis [26].

Song et al. [27] designed a fuzzy-based framework to classify COVID-19 patients using lung CT image analysis. First, a deep neural network was employed to extract low-level features. To enhance recognition performance, an attribute reduction algorithm was applied to derive more discriminative features. Then, key features were chosen to feed the fuzzy diagnosis model. Finally, different images in two classes were performed to test the trained fuzzy classifier [27].

Patel and Kashyap [28] developed an innovative approach based on two-dimensional wavelet transform and statistical features for COVID-19 detection in lung CT scan images. First, statistical features were extracted in four sub-bands of wavelet transform output. Next, PCA was performed to extract a subset of efficient features. Also, the extracted features were ranked based on their relation with class labels using Student’s test method. Finally, a least-squares SVM was used for classification [28]. Some researchers have explored innovative approaches to combine deep networks with handcrafted features with creative ideas. For example, Attallah [29] trained three versions of deep ResNet using two textural images derived from CT lung scans including a wavelet transform image and a gray level co-occurrence matrix. Features were then extracted from each trained network, combined, and normalized during the fusion process. Finally, an SVM was used for classification [29].

Kaushik et al. [30] introduced a three-layer stacked multimodal framework designed for deep feature extraction from a large dataset of COVID-19 chest radiographic images. Their approach employs eight transfer learning models, pre-trained on ImageNet, which are assessed using key performance metrics. A specialized stacking model then combines the outputs of these pre-trained networks, leveraging a three-layer architecture to extract deep features. These features are flattened, concatenated, and processed through seven dense layers with varying kernel and bias dimensions to optimize classification accuracy.

Liu et al. [31] presented a disease detection method with a low false positive rate (FPR) for COVID-19 lung images, introducing a dual-phase optimization framework. In the first phase, the model is trained using conventional gradient descent to extract relevant features. The second phase involves formulating an objective function aimed at minimizing the FPR, ensuring high accuracy while reducing FPR. This phase employs an evolutionary algorithm to fine-tune the model by selectively updating only the fully connected layer’s weights while keeping other parameters frozen. Through this two-stage optimization process, the method produces a deep learning model with enhanced diagnostic reliability and a low FPR.

Diagnosing pandemic diseases like COVID-19 is a multi-sectoral issue, and development and research in each sector can impact the accuracy of diagnosis and reduce mortality rates. Memos et al. [32] proposed a smart monitoring system combining emerging technologies (IoT, wireless sensor networks, big data, cloud computing, machine learning, and 5G networks) to enhance infectious disease detection and prevention, particularly for COVID-19. This integrated approach aims to modernize healthcare systems by combining cutting-edge technologies for faster, more efficient disease control. Some benefits of this system are automatic detection, reducing human intervention, speeding up diagnosis and preventing virus spread by isolating infected individuals, and enhancing accuracy through AI-driven big data analysis.

As previously explained, COVID-19 is a multifaceted disease that affects different parts of the body. Coughing is one of the common symptoms in most infected patients. Rashid et al. [33] present a scalable multifaceted DNN framework for detecting symptomatic cough of COVID-19 called CoughNet-V2. Their proposed framework was designed to help the specialists or doctors at the pre-screening stage for COVID-19 detection. In order to train CoughNet-V2, a crowd-sourced multimodal data resource that contains subjects’ cough audio along with some relevant medical records was collected. The reported results in [33] prove multimodal integration of cough audio along with medical records improves classification accuracy compared to some unimodal frameworks. A brief overview of related studies in COVID-19 diagnosis using X-ray and CT scan images is shown in Table 1.

## 3. Materials and Methods

As mentioned in the Introduction Section, beyond affecting the lungs’ visible appearance, the COVID-19 virus also directly impacts lung texture. Therefore, spatial domain analysis alone is insufficient for extracting discriminative features. Thus, the image texture requires specialized analysis. Machine-learning-based methods usually have two separate phases of feature extraction and classification, whereas deep learning-based networks perform feature extraction during the network’s training process. Thus far, many operators have been presented for image texture analysis, but nearly all of them are defined in the spatial domain, and using them in a deep learning-based structure is a serious challenge. In some studies, to solve this challenge, the input image is analyzed separately in two distinct phases using texture analysis operators and a deep network. The features extracted in each phase are then concatenated and fed into a supervised classifier. Research results have shown that this strategy for combining texture features and deep neural networks is inefficient for the following reasons.

- There is an imbalance between the number of features extracted using deep convolutional networks versus texture analysis operators and a much longer execution time of training and feature extraction in the deep network than using statistical texture analysis operators.
- The deep network needs to be trained, while in the other phase the texture operators are not trained.

**Figure 2:**
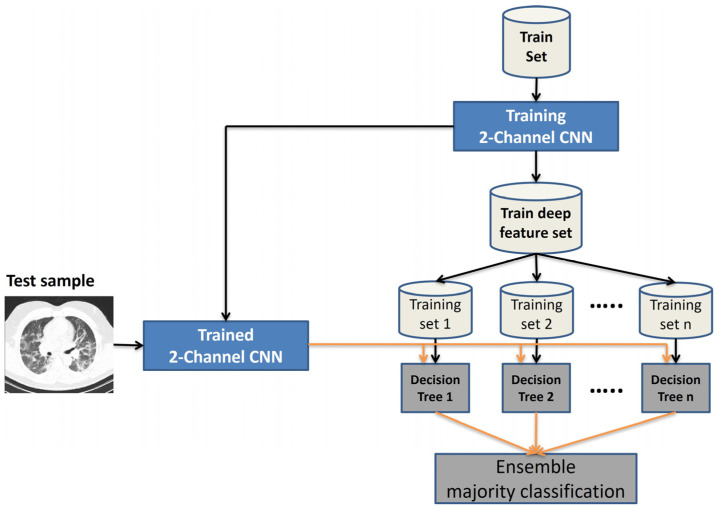
The block diagram of the proposed COVID-19 diagnosis system in terms of main steps.

This article presents a COVID-19 diagnosis method combining machine-learning and deep learning strategies. The presented method consists of three steps, preprocessing, feature extraction, and classification. The main structure of the proposed approach is shown in Figure 2. Each step is explained in detail in the following sections.

### 3.1. Preprocessing Step

Lung CT scan images are usually acquired by imaging devices with different brands and different qualities. Therefore, the acquired image quality in some laboratories may not be suitable. Therefore, the main goal in the preprocessing stage is to enhance the image quality and reduce noise. As mentioned above, the main innovation of this paper is the inclusion of texture features of lung CT scans in the diagnostic process. Therefore, the input of the first channel of the proposed deep CNN is the original image in the spatial domain. Also, the input of the second channel is the texture features in a matrix format of the same size as the original image. Therefore, using a single method to enhance the image quality of both channels is not efficient. To improve the quality of the original image, the histogram equalization algorithm is used as a simple and fast image enhancement method. Histogram equalization is a simple algorithm for adjusting image intensities to enhance contrast and brightness. Histogram equalization is a straightforward technique that adapts to the input image and its operator. In theory, the original input image can be recovered using the output histogram equalized image. The calculation is not computationally intensive.

- Histogram equalization algorithm can be performed in the following three steps:
- Build the histogram of the input image;
- Compute the normalized summation of histogram bins;
- Transform the input image to an output image.

The BM3D filter is used to enhance the contrast and reduce the noise in the second channel. The BM3D filter [36] is a well-known technique for suppressing adaptive white Gaussian noise (AWGN). BM3D is not originally designed for texture images, but research studies show that BM3D provides high performance in many cases such as texture image classification. Additionally, various improvements have been presented for BM3D including spatially correlated noise. The BM3D filter performs two different noise-reduction mechanisms [36] jointly. First, the patches (blocks) most similar to each given reference block are detected. Then, these blocks are collected to create a 3D array. Finally, the output 3D array is transformed into the spectral domain using separable two-dimensional DCT transform and a 1D-vertical Haar transform.

The modified BM3D (MBM3D) was first proposed by Rubel et al. to reduce spatially correlated noise [37]. MBM3D differs from the original BM3D in two aspects. First, the Bray–Curtis distance is applied for the similarity search as follows:

(1) $$d\left(Q,P\right)=\frac{\sum_{i=1}^{N}\left|Q_{i}-P\right|}{\sum_{i=1}^{N}\left|Q_{i}+P\right|}$$

where *P* shows the features of reference patch, *Q* means the feature vector of query image. *N* represents the total number of extracted features. DCT coefficients are used with *N* = 64 in this paper. Next, frequency-dependent thresholds are computed using Equation (2). In most research studies, *β* = 2.6 or slightly smaller provides the best performance.

(2) $$T\left(K,l\right)=\beta \sigma_{0}\sqrt{W_{norm}\left(K,l\right)}l=0,1,\ldots ,7$$

### 3.2. Feature Extraction Step

The method presented in this article is designed based on a machine-learning strategy. Therefore, the feature extraction step is performed independently from the classification step. As mentioned above, deep features are extracted through two separate channels in the deep convolutional neural network. The block diagram of our proposed approach is shown in Figure 2. The input of the first channel is the original image in the spatial domain, and the input of the second channel is the image texture feature matrix. To extract texture features, a modified LBP operator is used.

#### 3.2.1. Modified Local Binary Patterns

The local binary pattern (LBP) operator was first proposed by Ojala et al. [38] for image texture analysis. The LBP operator identifies common repeating patterns in small image neighborhoods by comparing neighbor intensities and assigning binary labels. First, a neighborhood with radius (R) is considered around each pixel of the image. Each neighbor’s intensity is then compared with the central pixel’s intensity. Next, a binary label (0 or 1) is assigned to each neighbor according to the following equation.

(3) $$s\left(i\right)=\left\{\begin{array}{c} 1\;\mathrm{if}\;i\geq 0\\ 0\;\mathrm{if}\;i<0 \end{array}\right.$$

Finally, by checking the completion of neighbors, a binary pattern with P bit is produced. By transferring this binary pattern to ten-format digits, a number is extracted as the LBP value of the central pixel (Equation (4)).

(4) $${LBP}_{P,R}(c)=\sum_{n=0}^{p-1}s(I_{n}-I_{c})2^{n}$$

For example, with 8 neighbors, the generated value ranges from 0 and 255. This process is applied independently to each image pixel. Ojala et al. [39] demonstrated that an LBP value histogram can effectively classify image textures with high accuracy. To reduce sensitivity to rotation, the neighborhood is usually assumed to be circular. Recently, many different versions of LBP have been proposed with the aim of extracting discriminative features. Ojala et al. [39] defined a uniformity measure “U” to categorize extracted binary patterns in a significant manner. The uniformity parameter U relies to the number of spatial transitions (bitwise 0/1 changes) in the extracted binary pattern. It is defined in Equation (5). For example, the uniformity of the pattern 01001100 is 4.

(5) $$\left({LBP}_{P,R}\left(x_{c},y_{c}\right)\right)=\left|S\left(I_{1}-I_{c}\right)-S\left(I_{P}-I_{c}\right)\right|+\sum_{n=2}^{P}S\left(I_{n}-I_{c}\right)-S\left(I_{n-1}-I_{c}\right)$$

Uniformity should be calculated for all of the extracted binary patterns. Next, patterns are grouped into two categories, “uniform” and “non-uniform”, based on a threshold UT. Hence, patterns with a uniformity value of less than UT are categorized as uniform. All patterns with a uniformity value greater than UT are known as non-uniform. Finally, a label is assigned to each pattern based on Equation (6). For uniform patterns, the label is assigned based on the total number of ones in their extracted binary pattern. The label value “*P*+1” is assigned to all of the non-uniform patterns.

(6) $$MLBP_{P,R}^{riu_{T}}=\{\begin{array}{ll} \sum_{k=1}^{P}(I_{k}-I_{c}) & \;\;\;\;\;\;\;\;\;\;\;if\;U(LBP_{P,R})\leq U_{T}\\ P+1 & elsewhere \end{array}$$

According to Equation (6), a numeric label in the range of [0–*P*] will be assigned to uniform patterns by applying LBP_P,R_. Also, the unique label “*P*+1” is assigned to all of the non-uniform patterns. In this respect, the U_T_ parameter should be optimized so that uniform labels cover most of the extracted binary patterns in the image. In most related research studies, the U_T_ parameter is chosen to be equal to *P*/4, meaning only a small fraction of texture patterns are detected as non-uniform. In *MLBP*, the relation between neighborhoods is not changed due to rotation, so this descriptor is invariant to rotation and gray scale variations. Image rotation disturbs the position of neighbors in relation to the center, but the relation between the neighbors will be the same after rotation. As mentioned above, *MLBP* extracts local texture information based on some predefined labels. In order to represent the texture as numerical features, a feature set vector can be extracted with “*P*+2” dimensions. First, *MLBP_P_*,*_R_* should be applied on the whole image and the labels assigned to neighbors. Then the occurrence probability of each label in the image is regarded as a feature. The occurrence probability of a specific label in the image can be approximated by the ratio of the number of pixels assigned that label to the total number of pixels (as per Equation (7)).

(7) $$=\left\{\frac{f_{0}}{N_{total}},\frac{f_{1}}{N_{total}},\frac{f_{2}}{N_{total}},\ldots ,\frac{f_{p+1}}{N_{total}}\right\}$$

where *f_i_* shows the number of neighbors (pixels) in the image with label *i*. Also, the *N_total_* shows the total number of neighbors which is same with image size.

#### 3.2.2. Proposed Deep CNN

**Figure 3:**
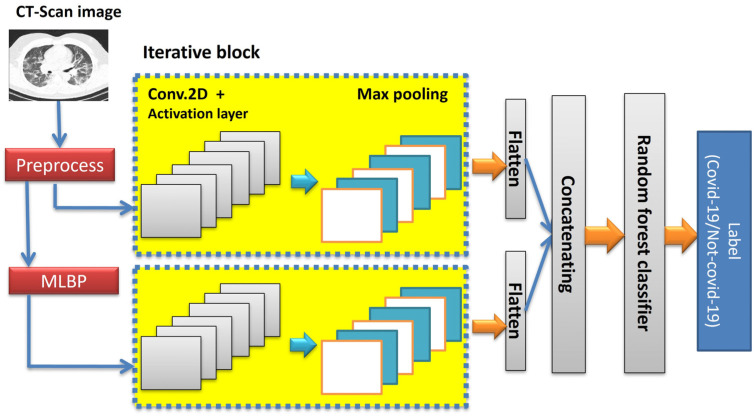
The internal structure of the proposed 2-channel CNN.

As shown in Figure 2, a deep convolutional neural network (DCNN) with two separate channels is used to detect COVID-19 in this paper. The input of the first channel is the original image whose quality has been improved in the preprocessing stage. The input of the second channel is the MLBP matrix, which is the same size as the original image. If the neighborhood radius in the MLBP operator is set to R, then by applying zero-padding of width R around the input image, the output of the MLP is a matrix of the same size as the original image. The size of the input images and the internal layers structure of both channels are considered the same. It decreases the total execution time of the process. This DCNN includes iterative block that can be repeated n times in sequentially. Iterative block consists of a two-dimensional convolution layer, swish activation function and a max pooling layer. The internal structure of the proposed 2-channel CNN is shown in Figure 3. The internal structure of the proposed 2-channel DCNN with 3 iterative blocks is shown in Table 2. In order to select trade-off between detection accuracy and runtime, an experiment is designed in Section 4.5.

The selection of the activation function in deep neural networks has a great impact on the final efficiency of the deep network and the dynamics of training. In most related articles, the ReLU (Rectified linear unit) activation function is usually used, which is shown in Equation (8).

F(x) = max (0, x)(8)

So far, different activation functions have been proposed, each of which has its own disadvantages and limitations. Therefore, the Swish function was presented for the first time by Google’s team and is defined in Equation (9).

F(x) = x·sigmoid(x) (9)

The test results provided by Google show that the Swish function works better than common functions such as the ReLU function in deeper models and challenging datasets. According to the Google report, replacing the ReLU function with Swish units improves the classification accuracy on the ImageNet dataset by 0.9% for Mobile NASNetA and 0.6% for the Inception-ResNet-v2 network. The Swish function is simple and can be easily replaced with ReLUs function units in any neural network. The Swish function provides this benefit along with being non-monotonic, which enhances the expression of input data and the weight to be learned. COVID-19 is still an unknown disease that produces a wide range of symptoms in different patients. Therefore, from the point of visual features, the lung CT scan of patients vary greatly from one another. Hence, we are faced with a challenging database in this article. For this reason, we have used the Swish activation function in the provided DCNN.

The structure of the proposed DCNN is defined dynamically in such a way that the user can change the number of iterative blocks. The results of our tests showed that repeating the blocks three times provides the highest accuracy among the possible numbers of repetitions in the range of 1 to 6. As mentioned in the introduction, the COVID-19 is a very new disease, and the number of samples in both healthy and diseased groups is not the same. The main contribution of this paper is to present a method for COVID-19 disease diagnosis based on a machine-learning strategy that utilizes deep features. Therefore, at the end of the proposed DCNNs, there are no classification layers such as fully connected or soft max. The output of both channels is connected to a flatten layer. A flatten layer collapses the spatial dimensions of the input into a single vector.

**Figure 4:**
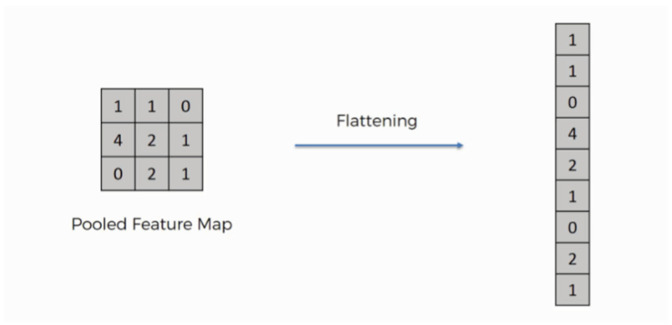
The process of the flatten layer.

The flatten layer is used to convert the two-dimensional matrix extracted from the internal layers of the deep network into a numerical feature vector (one dimensional array). The output of the flatten layer is used as input data to train the classifier in the proposed structure. The process of the flatten layer is shown with an example in Figure 4. Next, the outputs of both DCNNs are connected to each other and used to train a random forest classifier.

#### 3.2.3. Hyperparameter Optimization

In this article, a DCNN with two parallel channels is designed to detect COVID-19, one of which is fed with image texture information and the other with image spatial domain information. To adjust the weights of these two channels, the benchmark COVID-CT-349 database was divided into two parts, training and testing sets. About 30% of the dataset samples (224 images) were selected as the training set. In order to balance the size of both classes (negative COVID-19 and infected with COVID-19 cases), 112 samples were randomly selected from each class. All remaining images (522 samples) were considered as the test set. The details of the databases used in the experiments are explained in Section 4.1.

**Figure 5:**
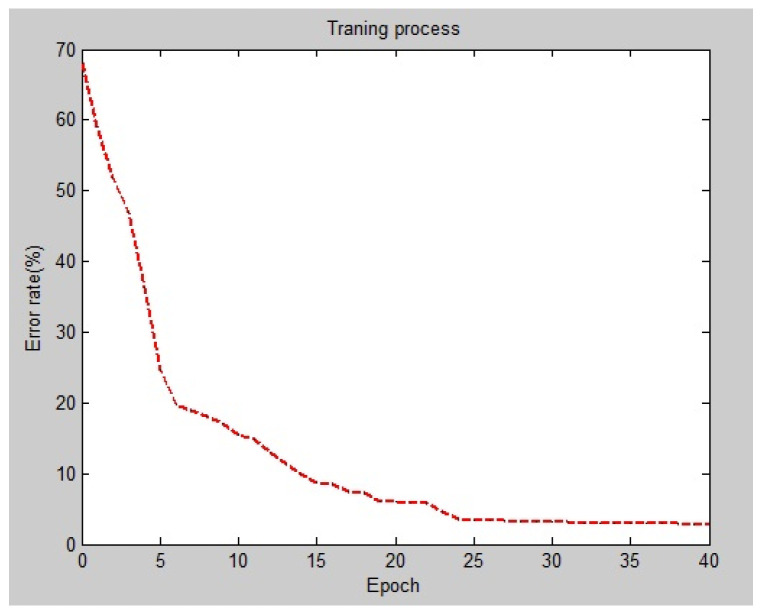
Error rate vs. epochs in the training process.

The Adam optimizer was used to optimize the hyperparameters. We also used a focal loss function with γ = 2 as the loss function for both of channels. To reduce the training runtime, both channels were trained for 40 epochs. First, initial learning rate is considered as 10^−5^ for 20 epochs. Next, to improve convergence, it was decreased to 10^−6^ for the next 20 epochs. A plot of the error rate versus the number of epochs during the training process is shown in Figure 5. Selecting a consistent and effective loss function plays an important role in CNN training. Multi-class cross-entropy is widely used in many studies. But, we are faced with an unbalanced two-class classification problem. Focal loss is another option, and we can leverage its properties to enhance our model’s performance. Focal loss applies a class weighting system to balance the samples in each batch size of data. It is shown in Equation (10).

FL(pt) = −αt(1 − pt)γ log(pt) (10)

Focal loss can be interpreted as a binary cross-entropy function multiplied by a modulation factor, (1 − pt)γ. The modulating coefficient reduces the contribution of easily classified samples. In Equation (10), pt is a function of real labels. Also, αt is a weighting factor that balances the effect of the moderating factor. Xavier initialization (Glorot) was also used to initialize the weights. Xavier is an advanced initialization scheme for CNNs. The biases and the weights were initialized in each layer (Equation (11)).

(11) $$W_{i,j}\sim U\left[-\frac{1}{\sqrt{S}},\frac{1}{\sqrt{S}}\right]$$

where U is a uniform distribution function, and S is the size of the previous layer.

The proposed DCNN was trained in 40 epochs. In order to select a more consistent learning rate and epoch values, our proposed DCNN was evaluated with a range of different possible values. The results are reported with details in Section 4.6. The learning rate was set to 10^−5^ for the first 20 epochs and reduced to 10^−6^ for the next 20 epochs. Also, a decay weight rate of 10^−2^ was considered for all epochs. This combination provided the highest accuracy, based on the results reported in Section 4.6.

### 3.3. Classification Step

As mentioned previously, the method presented in this article is based on a classical machine-learning structure. Therefore, the classification stage is separate from the feature extraction stage. For this reason, a variety of classifiers can be used at this stage. In this article, several different classifiers were tested; the random forest classifier provided the highest performance. The results of testing different classifiers are reported in the next section.

Random forest is a supervised machine-learning algorithm widely used for both classification and regression problems. It builds multiple decision trees on different data samples and uses their majority vote for classification or their average prediction for regression.

Random forest (RF) is a supervised ensemble learning method which can be used for classification. RF is made up of a set of low-depth decision trees. A decision is made in RF by constructing a multitude of decision trees during the training process and taking their majority vote. The main contribution behind random forest learning is that making a decision based on combining the decisions of several uncorrelated models, such as decision trees, performs better than them making decisions on their own. Two initial parameters, the number of trees and the maximum depth, can be used to tune random forest. Some of the advantages of random forest are as follows:

- In addition to classification, it can also be used for regression;
- Due to the concept of branches and trees, random forest predictions are more human-understandable than other classifiers;
- It can handle large datasets more efficiently than some other supervised classifiers;
- The random forest algorithm provides a higher level of accuracy in predicting outcomes than the decision tree algorithm.

## 4. Experimental Results

### 4.1. Datasets

The COVID-19 virus is a relatively new and widespread virus. Since the outbreak of this disease, various databases of CT scan images of patients’ lungs have been prepared all over the world. Therefore, to analyze and compared the efficiency of diagnostic methods in this field, they must be evaluated on the same database. For this purpose, in this article, we have evaluated the efficiency of the proposed method on a benchmark dataset. Also, the performance of the proposed method has been compared only with articles that have reported their efficiency on the same database.

**Figure 6:**
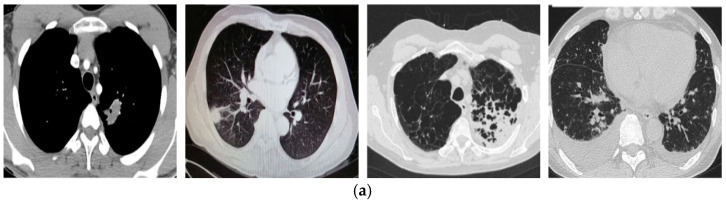

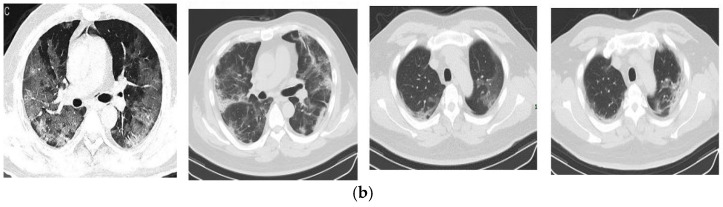
Some samples of COVID-CT-349 dataset. (**a**) Non-COVID-19 cases, (**b**) COVID-19 patients.

Zhao et al. [40] collected an open-source dataset of lung CT scan images in two classes, COVID-19 patients and non-COVID-19 cases, which is known as COVID-CT-349. This dataset contains 746 CT images from 349 patients labeled as positive for COVID-19, and 397 images labeled as negative for COVID-19. For each CT image, meta information is provided, which includes some properties such as patient age, gender, location, medical history, scan time, and severity of COVID-19. The CT images are not the same in size. The minimum and maximum height are 153 and 1853. Also, the minimum and maximum width are 124 and 1485 (Figure 6). As mentioned in Section 3.2.3, to adjust the weights of the proposed deep network, the COVID-CT-349 database was divided into two subsets: train and test sets. About 30% of the dataset samples (224 images) were selected as the training set and about 70% of samples (522 images) were used as the test set.

Also, in order to evaluate the generalizability of the proposed approach, another benchmark dataset from Italian Society of Medical and Interventional Radiology [41] is used. The Italian dataset included 100 axial CT scans from 60 COVID-19 patients. Another segmented CT scan dataset with annotation labels from Radiopaedia was added on 13 April 2020, further increasing the number of slices. In this study, 437 CT scans of this dataset were used.

### 4.2. Performance Evaluation Metrics

Considering the low transmission and mortality rates of common respiratory diseases such as the cold and flu, distinguishing patients with COVID-19 from other people (whether healthy or with other respiratory diseases) is the main goal of this article. Therefore, we are facing a two-class problem.

(12) $$\mathrm{Accuracy}=\frac{TP+TN}{TP+TN+FN+FP}$$

where TN and TP denote true negative and true positive, respectively. Also, FP and FN denote false positive and false negative. In this problem, the true positive represents the number of CT images that the person infected with the COVID-19 virus has been captured and identified as a COVID-19 virus-infected person by the proposed system too. The transmission rate of the COVID-19 virus is much higher than many other respiratory viruses. Therefore, to prevent the spread of the virus in the community, the cost of misclassifying an infected person as a healthy case is much higher than misclassifying a healthy sample as infected. Precision criteria focus on the TP rate more than accuracy, which can be used to evaluate the performance of unbalanced risk-based classification problems.

(13) $$\mathrm{Precision}=\frac{TP}{TP+FP}$$

### 4.3. Performance Evaluation of the Proposed Approach in Terms of Different Classifiers

As described above, the proposed approach follows a machine-learning strategy. Thus, it is possible to perform different classifiers in the classification phase. The performance of the presented method is evaluated using different classifiers as reported in Table 3. As can be seen in Table 3, the random forest (RF) provides the highest classification accuracy.

Random forest (RF) and support vector machine (SVM) are both powerful machine-learning algorithms, but they excel in different scenarios. RF is generally preferred for large, high-dimensional datasets and when interpretability is important, while SVMs can be more effective for smaller, well-structured datasets with clear separation boundaries. Also, in our proposed method, 6400 features are extracted using deep networks which is a high-dimensional set. Some benefits of RF are as follows: handles large datasets and high dimensionality, robust to outliers and missing data, feature importance, less prone to overfitting, interpretability, speed, and parallelism [42].

### 4.4. Random Forest Tuning

Random forest is a parametric classifier; if its parameters are tuned, the classifier’s efficiency can be increased. Maximum depth (D) and number of trees (N) are two main parameters in random forest that can be tuned. To find the optimal values for these two parameters, metaheuristic algorithms could be used. But it is extremely time-consuming and would reduce the generalizability of the method for real-world laboratory applications. Therefore, in this article, the ranges of allowed values for maximum depth [1,2,3,4,5,6,7] and number of trees [10,11,12,13,14,15,16,17,18,19,20,21,22,23,24,25,26,27,28,29,30,31,32,33,34,35,36,37,38,39,40] were divided into four equal intervals. Then the efficiency of the proposed method was evaluated for all 16 possible cases, the results of which are shown in the table below. As can be seen in Table 4, the combination *D* = 3 and *N* = 30 provides the highest accuracy about 94.63 percent. As can be seen in Table 4, the classifier’s efficiency decreases when the number of trees is increased to 40 and maximum search depth to 7.

COVID-19 diagnosis is a binary classification problem where the risk of misdiagnosis is not equal for both classes. As described above, the transmission rate of the COVID-19 virus is high; hence, the risk of misdiagnosis of a patient as healthy is much higher than in other cases. In this respect, more than precision, the confusion matrix of the classification process is shown in Table 5.

### 4.5. DCNN Optimization in Terms of Internal Blocks

**Figure 7:**
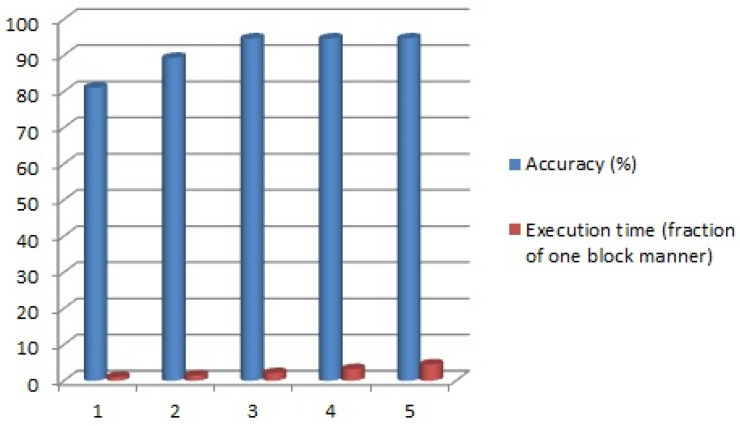
The performance evaluation based on different number of iterative blocks in terms of accuracy and execution time.

As explained in Section 3.2.2, the number of feature extraction iterative blocks in the proposed two-channel deep CNN can be determined by the user. In this experiment, the efficiency of the proposed method was evaluated based on different values for the number of iterative blocks from 1 to 5. The detection accuracy results are reported in the blue columns of Figure 7. Also, the execution time of the proposed method was evaluated for different numbers of iterative blocks. In this respect, the execution time of the proposed CNN with one iterative block was considered as the origin time, and the execution time of the two- to five-block modes compared to the origin time. The relative execution times are reported in the red columns in Figure 7.

**Figure 8:**
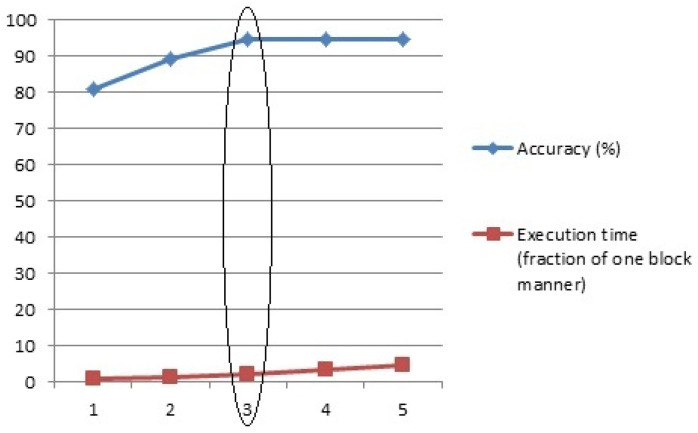
Trade-off between execution time and accuracy in terms of number of iterative blocks.

As can be seen, by increasing the number of blocks up to three, the accuracy of the proposed method increased rapidly, and after that, the growth acceleration decreased drastically and the accuracy increased by just 0.01% in a five-block manner. However, the execution time of the proposed method increased rapidly as the number of blocks increased. Therefore, in order to maintain the balance between detection accuracy and execution time, the effectiveness of the proposed method has been considered in three-channel mode (Figure 8).

### 4.6. DCNN Optimization in Terms of Learning Rate

As described above, training the proposed DCNN for 40 epochs provides maximum classification accuracy. In order to select a more consistent learning rate value and epoch number, our proposed DCNN was evaluated in terms of different possible values. The results are reported in Table 6. As can be seen in the table below, reducing the learning rate to 10^−6^ drastically decreased the classification accuracy.

### 4.7. Comparison Proposed Approach with Base-Line Methods

As mentioned in the Introduction, one of the main contributions of this paper is the use of deep features in a classical machine-learning-based structure to diagnose COVID-19 patients. In this experiment, the efficiency of the proposed method is evaluated once only based on the textural features extracted by the MLBP operator and tuned random forest. Its results are shown in Table 6. The second main contribution of this paper is to present a two-channel deep convolutional neural network, where one channel is fed with image spatial information and one channel is fed with texture features. In this experiment, the efficiency of the proposed method using just one channel of the proposed DCNN has been evaluated, and the results are shown in Table 7.

As mentioned above, one of the achievements of this research is the use of deep features in a classical machine-learning-based structure. In the scenario where the proposed DCNN without RF is considered, the same softmax layer that was used as a final layer to train the network also played the role of a classifier and the test data on the trained network were evaluated. In other words, in this scenario, the performance of the RF is compared to a classical softmax layer that is responsible for classification in DCNNs. As reported in Table 7, the use of the RF, due to a secondary training step within the tree structure, detects COVID-19 more accurately than the classically trained DCNN. These results demonstrate our claim about the advantages of using deep features in a machine-learning-based structure.

### 4.8. Comparison with State-of-the-Art Methods

The main goal of this article was to provide a method to diagnose the disease of COVID-19 more accurately than the existing methods. Therefore, in this experiment, the efficiency of the proposed method has been compared with some of the most efficient methods in this field under the same conditions in terms of the database and evaluation metrics. Results are reported in Table 8 and Table 9. Some articles have not declared the efficiency of the method based on the precision rate. Therefore, in Table 8, the term “NR” means “not reported”.

As can be seen in Table 9, the accuracy of the proposed method is higher than all the compared methods. The diagnosis accuracy of the method in [48] is 0.02 less than the proposed method. Also, the recall rate of the method in [48] is 0.26 less than our proposed method. The higher recall rate of our proposed method means that our model returns most of the relevant results. In [48], an architecture based on the U-net is presented, which has 10 convolutional layers in the encoding phase and 10 fully connected layers in the decoder structure, while the deep network architecture proposed in this paper has only four convolutional layers for the same input size. Therefore, the execution time of the proposed method is definitely less than in [48], which is another advantage of the proposed method over that in [48].

As reported in the results, the accuracy of the proposed method on the Italian database is higher than that on the COVID-CT-349 database. The ratio of COVID-19 to non-COVID-19 samples in the Italian database is 46.4:53.6 percent and in the COVID-CT-349 database, 46.7:53.3 percent. Therefore, both databases are only slightly unbalanced and do not differ much from each other in terms of data balance. Therefore, the reason for the superiority of the proposed method on the Italian database is not limited to database balance or overfitting. The dimensions of the samples in the COVID-Ct-349 database vary greatly. The height of the samples ranges from 153 to 1853 and the width of the images varies from 124 to 383. Therefore, in the preprocessing stage, in order to train the model, the dimensions of all the samples must be the same. Image resizing can lead to the loss of information or the creation of unrealistic pixels, while the dimensions of the images in the Italian database are all 512 × 512, resulting in fewer constraints during the resizing stage. This is one of the reasons for the better performance of the proposed method on the Italian database.

### 4.9. Offline and Online Process

As explained, the proposed model was trained in 60 epochs. Given the available hardware capabilities, training each channel of the proposed model took approximately 4.8 h. The training of deep neural networks is performed offline and has no direct impact on the end user. In other words, the system is trained offline only once by the development team. Then, users, including doctors and specialists, receive a response through a fully online process by providing a patient’s image to the trained system. Inference is an online process that plays a crucial role in clinical deployment. The evaluation results show that the decision time of the proposed model for a test image with dimensions of 256 × 256 is less than 5.3 s. Therefore, the results indicate that our proposed approach can make decisions in real applications.

## 5. Discussion

As discussed above, two main contributions are pursued in this paper. The first contribution was as follows:

- Using a two-channel CNN with the same architecture in channels and two different power sources provides better results for COVID-19 detection compared to existing one-channel methods.

The results reported in Table 7 showed that if the hyperparameters are well adjusted in a deep two-channel CNN, it can provide better detection accuracy than using a classical one-channel CNN. The second contribution of this manuscript was as follows:

- Using deep features in combination of supervised classifiers in which feature extraction and classification are performed separately achieves higher diagnostic accuracy than many popular deep CNNs for COVID-19 diagnosis.

Most of the methods compared in the Table 7 and Table 8 use deep neural networks. In these methods, the classification task is performed by the final layers in the deep neural network. The comparative results show that the extraction of deep features and the use of a supervised classifier (the proposed approach) provide better results for diagnosing COVID-19 cases. The third contribution of this article was that the COVID-19 virus, in addition to changes in the appearance of the lung, can also cause changes in the appearance of the lung texture. The input to the first channel of the provided deep CNN was the original image, while the input to the second channel was image texture information. As reported in Table 7, our experiments showed that the second channel of the proposed deep network, which was only fed with information of local texture changes, could accurately identify 91.86% of patients with COVID-19 separately. These results clearly show that texture changes include a large part of the changed characteristics in the lung images of COVID-19 patients compared to healthy patients.

In this paper, the efficiency of the proposed method was evaluated on two databases with a total of 1183 images. Thus, the number of samples and the diversity of imaging laboratories are limited. Therefore, training the system with a larger and more diverse set of samples would increase the model’s robustness to environmental variations.

Thus, it can be acknowledged that the proposed model was trained only on variants of the COVID-19 virus for which images were available in the databases. However, we know that this virus has the ability to mutate and produce new variants. Therefore, one of the limitations of the experiments is the dependence on the virus’s variants in the patients tested.

Random forest is an ensemble learning technique that enhances prediction accuracy and stability by combining multiple decision trees. It is applicable to both classification and regression tasks. The process consists of the following four key steps: bootstrap aggregating (bagging), feature randomization, individual tree construction, and prediction Aggregation. In the third step, a decision tree is trained for each bootstrapped and feature-randomized subset of the data. These trees are usually grown to their maximum depth without pruning. For the classification tasks, each tree independently predicts the class label for a new data point. The final output is determined by majority voting—the class with the most votes across all trees becomes the model’s prediction. Our results show that trees constructed using only features from the second channel (texture information) achieved approximately 9% higher accuracy in correctly labeling the test data than those using only features from the first channel (spatial domain).

The higher recall rate of the proposed approach, compared to the methods analyzed in Table 8 and Table 9, indicates its superior ability to identify more positive instances and minimize false negatives. A false negative in COVID-19 detection means a test result incorrectly indicates a person is not infected with the virus, even though they are actually carrying it. This can happen with both rapid antigen tests and PCR tests, though the reasons may differ. Therefore, given the high community transmission rate of COVID-19, a key advantage of the proposed method, in addition to high diagnostic accuracy, is its reduction in the false-negative rate. This can alleviate pressure on medical infrastructure and reduce the possibility of community transmission. In the context of COVID-19 detection, reducing false negatives means minimizing the chance that a test incorrectly indicates a person is not infected when they actually are. This is crucial for preventing the spread of the virus, as individuals who wrongly believe they are negative may not take necessary precautions. 

As shown in Table 8, the proposed deep fuzzy model in [27] achieves an accuracy of 94.2% and an F-score of 93.8%, which are 0.43% and 0.45% lower, respectively, than those of our method. Table 8 and Table 9 further demonstrate that our method outperforms all compared approaches in terms of F-score efficiency. The F-score, ranging from 0 to 1, serves as a key performance metric—higher values indicate better model effectiveness. In machine learning, the F-score is particularly useful when balancing precision and recall. A high F-score in classification reflects a model’s ability to accurately identify positive cases while minimizing both false positives (FPs) and false negatives (FNs). In the context of COVID-19 testing, reducing FPs (incorrectly diagnosing a healthy person as infected) and FNs (failing to detect an actual infection) is critical. Minimizing these errors helps to reduce disease transmission and optimizes patient management in healthcare facilities.

## 6. Conclusions

The main goal of this article was to present an efficient method for diagnosing COVID-19 patients through analysis of lung CT scan images. In this regard, a method including three stages of preprocessing, feature extraction, and classification was presented. The general structure of the proposed method is designed based on classical machine-learning techniques, with the difference that deep features are used in the feature extraction stage. For feature extraction, a deep convolutional neural network with two channels is proposed. The power source of one of the channels is the texture features of the CT scan image and the power source of the second channel is the observable information of the image in the spatial domain. The results of the experiments showed the following advantages in relation to the presented method:

- COVID-19 causes detectable lung texture changes. The proposed DCNN without the presence of a channel that feeds with image texture features provided a lower detection accuracy than the two-channel mode.
- Replacing deep CNN classification layers with supervised classifiers improved the COVID-19 diagnosis accuracy.
- Unlike methods that concatenate deep features with texture information, in this paper, the texture features were used to feed one of the channels of a deep convolutional neural network. The results showed that the presented two-channel CNN provides higher accuracy than many compared methods.
- Results show that COVID-19 disease, in addition to appearance properties such as color and shape, may disturb the texture of the patient’s lungs.
- The proposed method is a general approach where none of its steps are dependent on the type of input image. Therefore, an idea for future research is to apply the presented model in other visual pattern classification problems. The classification task in the proposed method originates from the trained two-channel deep neural network that is trained by spatial information and texture features of CT scan images. Therefore, the proposed two-channel deep neural network has a general architecture and can be used in similar classification problems such as diagnosing various lung diseases, diagnosing breast cancer, and diagnosing malignant tumors, in which texture variations play an important role.
- Like other viruses, COVID-19 continually evolves and undergoes mutations, resulting in new variants with distinct characteristics such as altered transmissibility and disease severity. Therefore, current detection methods, including ours, are inherently limited to recognizing variants present in their training data. However, our proposed method’s trainable architecture allows for adaptation to emerging variants by incorporating new CT scan images from patients infected with novel COVID-19 variants.

## Figures and Tables

**Table 1:** An overview on related studies.

Ref.	Year	Method	Classification	Advantages	Limitations
[24]	2021	Haralick features + pre-trained networks (VGG16, ResNet50, InceptionV3)	Deep learning	Lower execution time than trained deep networks	Lack of deep network training Lower performance than newer methods
[25]	2021	GLCM + LBP + MLP	Machine learning	Lower computational complexity than deep method	Lower performance than trained deep networks
[26]	2021	Handcrafted features (GLCM + LBP + SFTA) + PCA + SVM	Machine learning	Lower computational complexity than deep-based methods	Lower performance than deep learning-based methods
[22]	2022	EfficientNet + KPCA + Ensemble classifiers	Hybrid	Higher performance than compared methods in reference	Higher tunable parameters than single classifiers
[23]	2022	Fusion of Deep networks (Resnet50/Inception V3/Efficientb7)	Deep learning	Higher performance than compared machine-learning-based methods	Higher execution time than single deep neural network-based methods
[27]	2022	Deep features + Fuzzy classification	Deep learning	Higher performance than compared networks in the reference	Dependence of variants diagnosis on the redefinition of fuzzy rules
[28]	2022	Statistical features + DWT + PCA + SVM	Machine learning	Lower computational complexity than deep-based	Lower performance than trained deep networks
[29]	2022	Texture (GLCM/Wavelet transform) + ResNet	Hybrid	Higher performance than compared deep methods in reference	Lower detection accuracy than newer methods
[34]	2024	Pipeline (LBP + DWT + CNN)	Hybrid	Higher performance than compared networks in the reference	High ratio of COVID-19 samples to non-COVID-19 sample Lower detection accuracy than newer methods
[35]	2024	Fine-tuned AlexNet	Deep learning	Higher performance than pre-trained Alex-Net and compared networks in the reference	Very high ratio of non-COVID-19 samples to COVID-19 samples More parameters than some benchmark deep networks
[30]	2025	Fusion of pre-trained deep networks	Deep learning	Lower complexity than trained deep network-based methods	Lack of deep network training
[31]	2025	Residual neural network + Evolutionary algorithm	Deep learning	Higher performance than classical deep learning-based methods	High execution computation time due to using genetic algorithm

**Table 2:** The structure of the designed CNN in one channel.

Block	Layer Type	Input	Output	Details
1	Conv.2D	128 × 128 × 3	128 × 128 × 3	3 Filters 5 × 5, Stride 1, Pad 2
	Activation	128 × 128 × 3	128 × 128 × 3	Swish
	Pooling.2D	128 × 128 × 3	64 × 64 × 3	Max pooling, Stride 1, Spatial extent 1
2	Conv.2D	64 × 64 × 3	64 × 64 × 20	20 Filters 5 × 5, Stride 1, Pad 2
	Activation	64 × 64 × 20	64 × 64 × 20	Swish
	Pooling.2D	64 × 64 × 20	32 × 32 × 20	Max pooling, Stride 1, Spatial extent 1
3	Conv.2D	32 × 32 × 20	32 × 32 × 25	25 Filters 5 × 5, Stride 1, Pad 2
	Activation	32 × 32 × 25	32 × 32 × 25	Swish
	Pooling.2D	32 × 32 × 25	16 × 16 × 25	Max pooling, Stride 1, Spatial extent 1
4	Flatten	16 × 16 × 25	6400	Flatten

**Table 3:** Performance evaluation based on different classifiers in terms of accuracy (%).

Approach	Accuracy	Precision	Recall	F-Score
Naïve bayes	90.79	88.63	91.56	90.07
C4.5 tree	90.87	89.52	90.29	89.90
SVM	94.25	91.89	95.78	93.79
KNN, K = 3	91.45	90.76	90.71	90.73
KNN, K = 5	93.37	92.79	92.40	92.59
KNN, K = 7	93.08	92.27	92.40	92.33
Random forest	94.63	91.63	97.04	94.25

**Table 4:** Performance evaluation based on different tuning sets for random forest on COVID-CT-349 dataset in terms of accuracy (%).

Number of Trees	Maximum Depth	Accuracy
10	1	87.53
	3	89.28
	5	91.87
	7	90.36
20	1	87.69
	3	90.45
	5	94.01
	7	92.95
30	1	88.02
	3	92.76
	5	94.63
	7	92.61
40	1	87.91
	3	92.37
	5	93.35
	7	92.07

**Table 5:** The confusion matrix of the proposed COVID-19 diagnosis method on COVID-CT-349 dataset.

	Label	COVID-19	Non COVID-19
Predicted			
COVID-19		230	21
Non COVID-19		7	264

**Table 6:** The performance evaluation based on different learning rates and weight decay values on COVID-CT-349 dataset.

Learning Rate (First 20 Epochs)	Learning Rate (Second 20 Epochs)	Weight Decay Value	Accuracy
10^−4^	10^−5^	10^−3^	92.35
10^−4^	10^−5^	10^−2^	92.28
10^−4^	10^−6^	10^−3^	91.38
10^−4^	10^−6^	10^−2^	92.54
10^−5^	10^−5^	10^−3^	93.61
10^−5^	10^−5^	10^−2^	93.76
10^−5^	10^−6^	10^−3^	94.31
10^−5^	10^−6^	10^−2^	94.63
10^−6^	10^−6^	10^−3^	94.01
10^−6^	10^−6^	10^−2^	94.21
10^−6^	10^−7^	10^−3^	92.61
10^−6^	10^−7^	10^−2^	92.82

**Table 7:** Comparison results with base line algorithms of the proposed method on COVID-CT-349 in terms of accuracy (%).

Approach	Accuracy	Precision	Recall	F-Score
MLBP + RF	89.76	89.23	86.36	87.77
Proposed DCNN (first channel without RF)	91.61	90.65	91.79	91.21
Proposed DCNN (second channel without RF)	90.72	89.36	91.45	90.39
Proposed DCNN (first channel with RF as classifier)	92.74	91.88	91.74	91.80
Proposed DCNN (second channel with RF as classifier)	91.86	90.41	92.08	91.23
Proposed approach	94.63	91.63	97.04	94.25

**Table 8:** Comparison results with state-of-the-art methods on COVID-CT-349 in terms of accuracy (%).

Approach	Accuracy	Precision	Recall	F-Score
ResNet-50 [27]	77.4	NR	NR	74.6
VGG-16 [27]	76	NR	NR	76
EfficientNet-B1 [27]	79	NR	NR	79
DenseNet-169 [27]	79.5	NR	NR	76
Fine-tuned DenseNet-169 [43]	87.1	NR	NR	88.1
Deep fuzzy model [27]	94.20	NR	NR	93.8
ResNet-18 [44]	90.42	88.24	91.71	89.43
CovidNet-CT [44]	90.48	NR	NR	NR
DRE-Net [45]	84.74	NR	NR	84
CFRCF + Gabor + RF [46]	76.68	NR	NR	74.26
CFRCF + EMAP + SVM [46]	66.37	NR	NR	61.14
LBP + RF [46]	69.96	NR	NR	65.64
Conventional CNN [46]	77.03	NR	NR	67.92
Teacher–student framework + data augmentation [47]	79.56	84.59	74.93	79.47
Residual neural network + evolutionary algorithm [31]	83.04	88.18	79.50	83.62
Proposed approach	94.63	91.63	97.04	94.25

**Table 9:** Comparison results with state-of-the-art methods on Italian dataset [37] in terms of accuracy (%).

Approach	Accuracy	Precision	Recall	F-Score
Deep encoder–decoder + MLP [48]	95.40	NR	93.1	NR
COVID-Net [49]	92.60	NR	NR	NR
ShuffleNet [50]	70.66	53.48	65.26	58.79
Multitask [51]	94.67	NR	96	NR
Proposed approach	95.42	98.17	93.36	95.70

## Data Availability

The datasets generated during and/or analyzed during the current study are available from the corresponding author on reasonable request.
